# Meningococcal carriage in children and young adults: a cross-sectional and longitudinal study, Iceland, 2019 to 2021

**DOI:** 10.2807/1560-7917.ES.2023.28.39.2300215

**Published:** 2023-09-28

**Authors:** Iris Kristinsdottir, Linda J Visser, Willem R Miellet, Rob Mariman, Gerlinde Pluister, Gunnsteinn Haraldsson, Asgeir Haraldsson, Krzysztof Trzciński, Valtyr Thors

**Affiliations:** 1 University of Iceland, Faculty of Medicine, Reykjavik, Iceland; 2 Children’s Hospital Iceland, Landspitali University Hospital, Reykjavik, Iceland; 3 Centre for Infectious Disease Control Netherlands, National Institute for Public Health and the Environment (RIVM), Bilthoven, The Netherlands; 4 Department of Pediatric Immunology and Infectious Diseases, Wilhelmina Children's Hospital, University Medical Center Utrecht, Utrecht, The Netherlands; 5 Department of Clinical Microbiology, Landspitali University Hospital, Reykjavik, Iceland

**Keywords:** Neisseria meningitidis, Meningococcal, Carriage, Whole genome sequencing, Adolescent, Young Adult, Non-typable

## Abstract

**Background:**

*Neisseria meningitidis* is a commensal bacterium which can cause invasive disease. Colonisation studies are important to guide vaccination strategies.

**Aim:**

The study’s aim was to determine the prevalence of meningococcal colonisation, duration of carriage and distribution of genogroups in Iceland.

**Methods:**

We collected samples from 1 to 6-year-old children, 15–16-year-old adolescents and 18–20-year-old young adults. Carriers were sampled at regular intervals until the first negative swab. Conventional culture methods and qPCR were applied to detect meningococci and determine the genogroup. Whole genome sequencing was done on groupable meningococci.

**Results:**

No meningococci were detected among 460 children, while one of 197 (0.5%) adolescents and 34 of 525 young adults (6.5 %) carried meningococci. Non-groupable meningococci were most common (62/77 isolates from 26/35 carriers), followed by genogroup B (MenB) (12/77 isolates from 6/35 carriers). Genogroup Y was detected in two individuals and genogroup W in one. None carried genogroup C (MenC). The longest duration of carriage was at least 21 months. Serial samples from persistent carriers were closely related in WGS.

**Conclusions:**

Carriage of pathogenic meningococci is rare in young Icelanders. Non-groupable meningococci were the most common colonising meningococci in Iceland, followed by MenB. No MenC were found. Whole genome sequencing suggests prolonged carriage of the same strains in persistent carriers.

**Key public health message**

**What did you want to address in this study and why?**

Meningococci are bacteria that young healthy people carry in their throat but have the potential of causing a life-threatening disease. Vaccines are available against some meningococcal types. Knowledge on how common carriage of meningococci is and which types are carried is essential to determine vaccination strategies.

**What have we learnt from this study?**

Meningococcal carriage is uncommon in children, adolescents and young adults in Iceland. Most carried so-called non-typable meningococci that are usually non-pathogenic. Meningococcal group B was the most commonly carried pathogenic group, carried by 0.7% of adolescents and young adults. None carried group C, which is the only group that is currently vaccinated against in Iceland.

**What are the implications of your findings for public health?**

Carriage of pathogenic meningococci was uncommon, probably due to effective vaccination schedules. Together with the low incidence of invasive meningococcal disease in Iceland, our results do not suggest the need for additional meningococcal vaccinations in the national immunisation programme.

## Introduction

*Neisseria meningitidis* is a common human upper respiratory tract commensal. Meningococci can cause invasive bacterial disease, with a rapid onset, severe manifestations and subsequent sequelae in survivors [1-3]. Six capsular groups of *N. meningitidis* cause the vast majority of invasive meningococcal disease (IMD): A, B, C, W, X and Y [4]. The prevalence of meningococcal colonisation peaks among adolescents and young adults and therefore, most colonisation studies focus on these age groups [5]. A monovalent MenC vaccine was introduced in the Icelandic immunisation programme in 2002 (in a two-dose schedule at 6 and 8 months of age), with a catch-up campaign for < 19-year-olds (one dose). Subsequently, the incidence of IMD decreased substantially in Iceland and has remained low. However, increased infection rates with groups W (MenW) and Y (MenY) have been observed in some European countries for the past years, for example the United Kingdom, Denmark and the Netherlands [6,7]. Knowledge on carriage is important for understanding of the epidemiology and transmission dynamics of *N. meningitidis*.

The study’s aims were to assess the prevalence of meningococcal colonisation among children, adolescents and young adults in Iceland and to ascertain the prevailing serogroups. Furthermore, we evaluated the duration of carriage in the longitudinal arm of this study and the relatedness of detected isolates.

## Methods

### Study design and participants

This was a descriptive point prevalence and longitudinal study of meningococcal carriage among Icelandic children, adolescents and young adults. Three age groups were included: (i) 1–6-year-old children attending daycare centres (DCCs), (ii) 15–16-year-old adolescents in the last year of lower secondary school and (iii) young adults aged 18–20 years attending college. Participants were recruited from 15 DCCs, five secondary schools and three colleges in the greater Reykjavik capital area. The DCCs and schools were selected by size and catchment area. All students at the schools were offered participation in the study. Recruitment was done and first swabs were collected in March and April 2019. Meningococcal carriers were invited for follow-up sampling at 3–6 month intervals until the first negative swab. Carriers did not receive antibiotics for decolonisation, as this is generally not recommended. Meningococcal vaccination history and data on filled prescriptions for antibiotics in the 30 days before the first swab, and between follow-up swabs for participants in the longitudinal part of the study, were retrieved from national electronic databases on vaccinations and medicine prescriptions governed by the Directorate of Health. These data were only collected for the adolescent and young adult groups.

### Sampling and laboratory methods

Nasopharyngeal swabs were obtained from participants in the youngest age group. Oropharyngeal swabs were collected from adolescents and young adults, with both tonsils/tonsillar beds swabbed with the same pin. All samples were collected using eSwab pins (Copan) and were immediately placed in 1 mL liquid Amies transport medium. Trained healthcare personnel collected the samples. Samples were plated on horse blood agar, chocolate agar and GC agar within 6 h from collection and incubated at 36 °C in 5% carbon dioxide. Plates were visually examined the next day for meningococcal growth. Meningococcus-like colonies were tested for oxidase reaction (MERCK). If positive, a single colony was cultured on chocolate agar (in house-made) to be tested with mass spectrometry (MALDI-TOF, Bruker). The swab pins with transport medium and the meningococcal strains were stored at −80 °C for later molecular diagnostics.

We extracted DNA from the Amies medium and also from the frozen strains, cultured on chocolate agar, for qPCR and whole genome sequencing (WGS), using a MagNA Pure system (Roche Molecular System).

For detection of meningococcal DNA, qPCR was done on culture- and mass spectrometry-positive samples. In addition, the same was done for every 10th sample collected at the first sampling time point classified as negative for *N. meningitidis* by culture. We used *metA* and *ctrA* as gene targets [8,9] for the detection of *N. meningitidis *and qPCR for serogroup determination using *csaB*, *csb*, *csc* [9], *csw* [6] and *csy* [9] as gene targets for groups A, B, C, W and Y, respectively. Samples with quantification cycle (Cq) values of < 40 were considered positive by qPCR. We assessed overall bacterial abundance by 16S qPCR [10]. The abundance of *N. meningitidis* relative to the overall bacterial abundance in the oropharyngeal swabs was calculated based on *metA* and 16S qPCR results.

We performed WGS on the 14 viable groupable *N. meningitidis* strains. We prepared DNA libraries using the Nextera DNA Flex Library Prep kit (Illumina), followed by paired-end sequencing (2 × 150 bp) on an Illumina NextSeq platform (Illumina). Read quality analysis and de novo assembly was done with the Juno-assembly v2.0.2 pipeline (https://github.com/RIVM-bioinformatics/juno-assembly). Briefly, read quality assessment and filtering were done using FastQC and FastP. Genomes were assembled using SPAdes and curated with QUAST, CheckM and Bbtools. Subsequently, isolates were typed with MLST v2.19.0 (https://github.com/tseemann/mlst). In silico capsule typing was performed as described by others [11] and cgMLST was done on pubMLST [12] using the Neisseria cgMLST scheme [13]. The cgMLST data were used to construct a minimum spanning tree with GrapeTree v2.1 [14]. The data are available on PubMLST (isolate IDs 123755–123768).

### Data analysis and statistics

Descriptive statistical analysis was done. We used chi-square test and Fisher’s exact test to compare categorical variables and Mann–Whitney U test for numerical variables. A p value of < 0.05 was considered statistically significant. Analysis was done in R version 4.2.2 (R Foundation).

### Definitions

Samples that were both culture-positive and positive for *metA* on qPCR from the oropharyngeal swabs or from a cultured strain were considered positive for meningococci. This was done to reduce the chances of counting commensal *Neisseria* spp. as *N. meningitidis*. Culture-positive samples that were either qPCR-negative or not available for qPCR were however considered positive for *N. meningitidis* if the MALDI-TOF score was ≥ 2. Samples positive for *metA* and *ctrA* were defined as positive for groupable meningococci, whereas positivity for *metA* alone was defined to represent carriage of non-groupable (NG) meningococci. Participants with more than one positive sample for any meningococci were defined as prolonged carriers. Non-prolonged carriers were defined as those from whom two consecutive samples were taken, of which the latest was negative.

## Results

We collected a total of 1,182 samples at recruitment to the study in the spring of 2019, 460 from children attending DCCs, 197 from adolescents and 525 from young adults, which represents about 20% of eligible participants (Table 1). The mean age was 3.7 years (standard deviation (SD): 1.2) for the DCC children, 15.8 years (SD: 0.28) for the adolescents and 18.9 years (SD: 0.59) for the young adults. Fifty per cent of the DCC children, 57% of adolescents and 61% of young adult participants were female. No children attending DCCs were identified as meningococcal carriers in the study. Meningococci were detected by culture in three adolescents (3/197; 1.5%) and 34 young adults (34/525; 6.5%). Only one of the three samples from the adolescents was also qPCR-positive for meningococci (1/197; 0.5%). The MALDI-TOF MS score was < 2 for both qPCR-negative samples and they were therefore defined as not meningococci. The number of positive samples remained the same for young adults by qPCR, resulting in a total of 35 meningococcal carriers.

**Table 1:** Enrolled participants, *Neisseria meningitidis* carriage, vaccination status and antibiotic use, Iceland, 2019–2021 (n = 1,182)

	Total		Carriers		Non-carriers		p value
	n	%	n	%	n	%	
**Age group**	1,182		35		1,147		
DCC children	460	38.9	0	0	460	40.1	< 0.001
Adolescents	197	16.7	1	2.9	196	17.1	
Young adults	525	44.4	34	97.1	491	42.8	
**Sex**							
Female	663	56.1	21	60.0	642	56.0	0.76
Male	519	43.9	14	40.0	505	44.0	
**College (young adults)**	525		34		491		
College A	165	31.4	12	35.3	153	31.2	0.29
College B	223	42.5	17	50.0	206	42.0	
**College C**	137	26.1	5	14.7	132	26.9	
**Adolescents and young adults only**	722		35		687		
Filled antibiotic prescription in the 30 days before sampling	36	5.0	1	2.9	35	5.1	1.0
Antibiotics effective against meningococci^a^	30	4.2	0	0	30	4.4	0.4^c^
Antibiotics not effective against meningococci^b^	6	0.8	1	2.9	5	0.7	
Meningococcal vaccinations	582	80.6	30	85.7	552	80.3	0.52
MenC vaccine	580	80.3	30	85.7	550	80.1	
MenACWY vaccine	6	0.8	0	0	6	0.9	
Monovalent meningococcal vaccine, unknown vaccine type	1	0.1	0	0	1	0.1	
No registered vaccinations	140	19.4	5	14.3	135	19.7	
**Adolescents only**	197		1		196		
Vaccinated adolescents	175	88.8	1	100	174	88.8	1.0
Monovalent MenC vaccine	174	88.3	1	100	173	88.3	
1 dose	23	11.7	0	0	23	11.7	
2 doses	150	76.1	1	100	149	76.0	
3 doses	1	0.5	0	0	1	0.5	
Monovalent meningococcal vaccine, unknown vaccine type	1	0.5	0	0	1	0.5	
Tetravalent MenACWY vaccine	2	1.0	0	0	2	1.0	
**Young adults only**	525		34		491		
Vaccinated young adults	407	77.5	29	85.3	378	77.0	0.39
Monovalent MenC vaccine	406	77.3	29	85.3	377	76.8	
1 dose	400	76.2	29	85.3	371	75.6	
2 doses	6	1.1	0	0	6	1.2	
Tetravalent MenACWY vaccine	4	0.8	0	0	4	0.81	

DCC: daycare centre.

^a^ Amoxicillin, amoxicillin-clavulanic acid, phenoxymethylpenicillin, doxycycline, lymecycline or azithromycin.

^b^ Clindamycin, cloxacillin or cephalexin.

^c^ Comparison of antibiotics effective against *N. meningitidis* vs non-effective antibiotics or no antibiotics.

We did not collect data on vaccinations for the DCC children but the general MenC vaccine uptake in the infant immunisation schedule was around 94% [15]. Eighty-one per cent (582/722) of adolescents and young adults had received at least one dose of a meningococcal vaccine, of whom 580 had been vaccinated with a monovalent MenC vaccine (Table 1). Of the adolescents, 88.8% (175/197) had been vaccinated against meningococci, the majority (76.1%) with two doses of a monovalent MenC vaccine according to the childhood immunisation schedule. Vaccinations were registered for 77.5% (407/525) of the young adults, 76.2% (400/525) were vaccinated with one dose of a monovalent MenC vaccine, according to the catch-up campaign (Table 1). Six of 722 adolescents and young adults (0.8%) were vaccinated with a tetravalent MenACWY vaccine, five of them had also been vaccinated with a monovalent MenC vaccine. One participant had received a monovalent meningococcal vaccination, not further specified (Table 1). There was no record of any of the participants being vaccinated with a MenB vaccine. Nineteen per cent of adolescents and young adults (140/722) had no registered meningococcal vaccinations, of whom five were meningococcal carriers (5/35 carriers; 14.3%). The other 30 carriers had been vaccinated against meningococci, all with a MenC vaccine and none with MenACWY. Carriage rates did not differ between vaccinated and unvaccinated individuals (p = 0.52).

Thirty-six adolescents and young adults (5%) had filled a prescription for antibiotics in the 30 days preceding the first sampling, including one carrier who filled a prescription for cloxacillin, an antibiotic not effective against meningococci. The remaining 35 who had filled antibiotic prescriptions were non-carriers; thirty of them had filled prescriptions for antibiotics that could potentially eradicate colonisation (Table 1).

**Figure 1:**
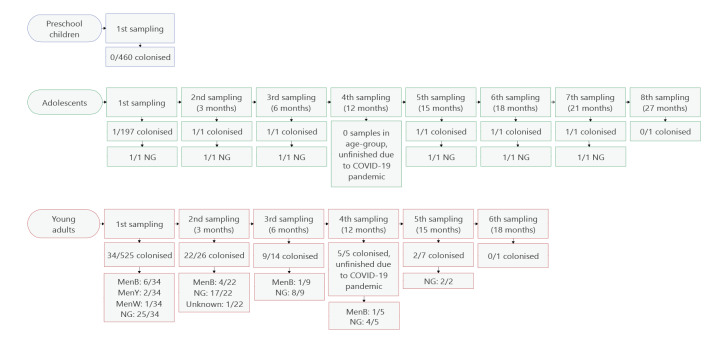
Overview of collected samples and samples positive for *Neisseria meningitidis*, Iceland, 2019–2021 (n = 1,182)

Follow-up swabs were obtained from carriers at intervals of 3–6 month. Figure 1 shows the number of positive samples among the collected samples at each follow-up sampling time point. Not all swabs could be collected at 12 months due to the COVID-19 pandemic, so all colonised participants at the 6-month sampling time point were invited back for the 15-month follow-up. All 22 participants with more than one positive sample subjected to qPCR carried the same meningococcal serogroup at each sampling time point (Table 2). One MenW carrier was lost to follow-up, and both MenY carriers had negative follow-up samples. One participant had a culture-positive follow-up sample with a MALDI-TOF MS score > 2, but the sample was not available for qPCR. The longest detected carriage of groupable meningococci (MenB) lasted at least 12 months (from the first positive to the last positive sample), and of non-groupable meningococci for at least 21 months (Figure 1, Table 2). No difference was observed in the absolute abundance (median: 7.54 × 10^–6^ ng/μL vs 9.14 × 10^–6^ ng/μL; p = 0.50) nor relative abundance (0.000087 vs 0.00107; p = 0.45) of meningococci in the first samples from prolonged vs non-prolonged carriers. Both absolute (median 5.58 × 10^–5^ ng/μL vs 7.58 × 10^–6^ ng/μL, p = 0.004) and relative abundance (median: 0.0005 vs 0.00006; p = 0.001) of meningococci were higher in follow-up samples than first samples.

MenB: *N. meningitidis* serogroup B. MenY: *N. meningitidis* serogroup Y. MenW: *N. meningitidis* serogroup W; NG: Non-groupable meningococci.

Shown is the number of participants at each sampling time point, the number of colonised participants and the number of participants with each serogroup by age group. One follow-up sample at the second sampling in the young adult group was not available for qPCR, and information about the serogroup is therefore not available and labelled unknown in the Figure.

**Table 2:** Follow-up of carriers of *Neisseria meningitidis*, Iceland, 2019–2021 (n = 35)

ID	**Baseline** **1st sample**	**3 months** **2nd sample**	**6 months** **3rd sample**	**12 months** **4th sample**	**15 months** **5th sample**	**18 months** **6th sample**	**21 months** **7th sample**	**27 months** **8th sample**
0005	NG	NG	NG	No sample^a^	Negative	NS		
0011	NG	NG	Negative	NS				
0022	NG	NG	Negative					
0025	NG	NG^b^	Negative					
0038	NG	NG	NG^b^	NG	Negative	NS		
0067	NG	NG	NG	NG	NG^c^	Negative	NS	
0074	MenY	Negative	NS					
0081	MenB	MenB^b^	Negative	NS				
0103	NG	NG	Lost to follow-up					
0107	NG	Lost to follow-up	NS					
0129	NG	Lost to follow-up						
0141	MenB	Lost to follow-up						
0169	NG	NG	Lost to follow-up	NS				
0174	NG	NG	Lost to follow-up					
0190	NG	NG	NG	No sample^a^	Negative	NS		
0191	NG	NG	Lost to follow-up	NS				
0251	NG	Negative	NS					
0252	NG	NG	Lost to follow-up					
0254	MenB	MenB	MenB	MenB	Negative	NS		
0258	NG^c^	NG^b^	Negative	NS				
0259	NG	NG^c^	NG	No sample^a^	Lost to follow-up	NS		
0267	NG	Lost to follow-up	NS					
0297	NG	NG^c^	NG	NG	Negative	NS		
0302	MenY	Negative	NS					
0305	NG	Culture-positive^d^	Lost to follow-up	NS				
0312	NG	Negative	NS					
0328	MenB	Lost to follow-up	NS					
0338	NG	NG^c^	NG^c^	NG	Lost to follow-up	NS		
0347	MenB	MenB	Lost to follow-up	NS				
0430	NG	NG	NG	No sample^a^	NG	Lost to follow-up	NS	
0450	NG	Lost to follow-up	NS					
0503	MenW	Lost to follow-up						
0504	MenB	MenB	Lost to follow-up	NS				
0536	NG	Lost to follow-up	NS					
0571	NG	NG	NG	No sample^a^	NG	NG	NG	Negative

ID: identification number; MenB: *N. meningitidis* serogroup B; MenY: *N. meningitidis* serogroup Y; MenW: *N. meningitidis* serogroup W; NS: no sample taken; NG: non-groupable.

^a^ Collection of swabs at the 12-month follow-up was unfinished due to restrictions in connection with COVID-19 pandemic.

^b^ Antibiotic use, effective against *N. meningitidis* (phenoxymethylpenicillin, amoxicillin, amoxicillin-clavulanic acid), following the sampling time point.

^c^ Antibiotic use, not effective against *N. meningitidis* (clindamycin, dicloxacillin, cephalexin and mecillinam), following the sampling time point.

^d^ Culture-positive but sample not available for qPCR.

The table shows the serogroups according to qPCR at each sampling time point during a follow-up of meningococcal carriers.

Eight of 23 persistent carriers filled antibiotic prescriptions between follow-up swabs, including four who filled prescriptions for antibiotics effective against *N. meningitidis* (phenoxymethylpenicillin, amoxicillin, amoxicillin-clavulanic acid). One of these four carriers had a swab positive for meningococci following the antibiotic treatment.

**Figure 2:**
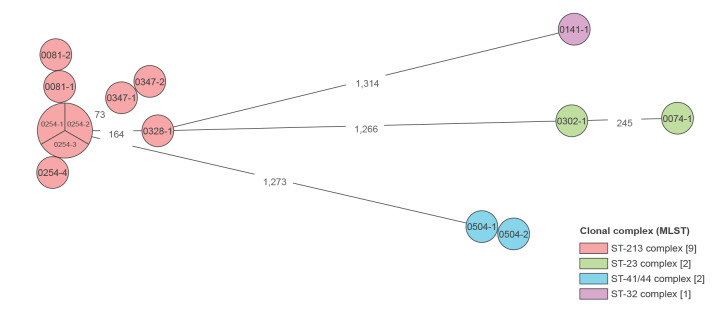
Relationship between capsulated *Neisseria meningitidis* strains, Iceland, 2019–2021 (n = 14)

CC: clonal complex; MLST: multilocus sequence typing; ST: sequence type.

Ten strains from four prolonged carriers were subjected to WGS. Seven of the 10 strains were identified as MenB and three as non-groupable with a B backbone, having phase variable off in the *csb* gene (Table 3). Strains from three of four prolonged carriers with capsulated meningococci were of sequence type (ST) 213 and clonal complex (CC) 213, and the strains from the fourth prolonged carrier were MenB ST-409 and CC-41/44 (Table 3). Strains from the same carrier were closely related, with 0–3 allelic differences (Figure 2). No allelic differences were detected between the first three strains from carrier 0254, which were of genogroup B. The fourth strain had one allelic difference from the other three (Figure 2) and was non-groupable with a B backbone (Table 3). Strains from two carriers (0081 and 0254) were separated by 3–5 allelic differences (Figure 2). The two participants were both college students but from different colleges, and no information was available on their social interactions. Furthermore, four *metA*/*ctrA*-positive strains from non-prolonged carriers were also sequenced (Table 3): they were MenY (two strains), MenB (one strain) and non-groupable with a B backbone, in which the *csb* gene was disrupted by an insertion element (one strain). One strain (MenW according to qPCR on the oropharyngeal swab) was non-viable and could therefore not be sequenced. The two MenY strains were both of CC-23 but were distinct STs, ST-23 and ST-1655. The MenB strain from a non-prolonged carrier was ST-213, the same ST and CC as the MenB strains from prolonged carriage. The ST-213 strains from different carriers had 3–171 allelic differences. The allelic differences between strains of different CCs were 1,260–1,317.

**Table 3:** Whole genome sequencing of capsulated meningococci, Iceland, 2019–2021 (n = 14)

Sample ID	Genogroup	Mutation in capsule locus	ST	CC
Prolonged carriage				
1–0081	NG – B backbone	Phase variable off in *csb*	213	213
2–0081	NG – B backbone	Phase variable off in *csb*	213	213
1–0254	B	None	213	213
2–0254	B		213	213
3–0254	B		213	213
4–0254	NG – B backbone	Phase variable off in *csb*	213	213
1–0347	B	None	213	213
2–0347	B		213	213
1–0504	B		409	41/44
2–0504	B		409	41/44
Non-prolonged carriage				
1–0074	Y	None	23	23
1–0141	NG – B backbone	*csb* disrupted by insertion element	11527	32
1–0302	Y	None	1655	23
1–0328	B		213	213

CC: clonal complex; NG: non-groupable; ST: sequence type.

Minimum spanning tree based on cgMLST showing the relationship between capsulated strains. Colours represent CC. Circles are labelled with sample identification numbers. The numbers on the branches represent the number of allelic differences. There were no allelic differences between strains 0254–1, 0254–2 and 0254–3; the three strains are represented with one bigger circle. One allelic difference separated strain 0254–4 from the other three 0254 strains. Six of the strains of CC ST-213 were identified as genogroup B (0254–1, 0254–2, 0254–3, 0347–1, 0347–2 and 0328–1), the other three (0254–4, 0081–1, 0081–2) as non-groupable with a B backbone. Both isolates of CC ST-41/44 were of genogroup B, the two of CC ST-23 of genogroup Y and the one CC ST-32 isolate was non-groupable with a B backbone.

## Discussion

In this point prevalence and longitudinal study, we describe the carriage of *N. meningitidis* in young children, adolescents and young adults in Iceland. No carriage was detected among children in daycare centres. Carriage rates in 15–16-year-olds and 18–20-year-old young adults were 0.5% and 6.5%, respectively. We report carriage of groupable meningococci for at least 12 months and of non-groupable meningococci for at least 21 months. Our work suggests that prolonged carriage of meningococci is due to persistent carriage of the same strain rather than re-colonisation with a different strain. This is, to our knowledge, the longest meningococcal carriage that has been reported.

Meningococcal carriage rates have frequently been reported to range from 15 to 25% in cohorts vaccinated against MenC [2,16]. Some recent carriage studies have reported lower carriage rates (6.2–7.2%), similar to our results in the young adult group (6.5%), which may be attributed to changes in social behaviour among teenagers and young adults [17,18]. The majority of carried meningococci were classified as non-groupable, as also described in other studies [19]. Of the groupable meningococci, MenB were the most prevalent in our study. This is in concordance with other European carriage studies [2,5].

No carriers were identified among the 460 preschool children in our study, which is less than expected. A previous study from the Netherlands reported a 3.2% colonisation prevalence among children in their second year of life [20]. In a meta-analysis, the prevalence of meningococcal carriage in infants was estimated at 4.5%, increasing nonlinearly to 7.7% in 10-year-olds and peaking in 19-year-olds at 23.7% [21]. Colonisation with* Neisseria lactamica*, which is most common among young children, is believed to be protective against colonisation with *N. meningitidis* [22,23]. The mechanism of the protection remains unclear, but competition between the two *Neisseria* species in the pharynx is hypothesised as a possible reason [23]. Although we did not specifically look for it, it is plausible that high carriage rates of *N. lactamica* could partly explain the absence of *N. meningitidis* among the preschool children in our study. Furthermore, as nasopharyngeal swabs, rather than oropharyngeal swabs, are preferred in preschool children, this may have influenced the sensitivity of meningococcal carriage detection [24]. High rates of antibiotic consumption among preschool aged children in Iceland may also contribute to a low prevalence of meningococcal carriage [25].

Eighty-eight per cent of the adolescents and 77.3% of the young adults were vaccinated with a monovalent MenC vaccine. The monovalent MenC vaccine had been added to the immunisation programme at the time when participants in the adolescent age group received their childhood vaccinations and has since led to a substantial reduction in meningococcal disease of all ages in Iceland. However, participants in the young adult group belong to birth cohorts that were included in the catch-up campaign, which might explain the lower vaccine coverage. Monovalent MenC conjugate vaccines have been shown to significantly reduce MenC carriage [26]. This was confirmed in our study as no MenC was found in the entire cohort. Studies show conflicting results regarding the effects of tetravalent MenACWY conjugate vaccines on the carriage of the vaccine serogroups, and MenB vaccinations (protein, or protein and outer membrane vesicle vaccines) do not seem to affect the carriage of disease-causing meningococci [26-28].

In this longitudinal study, we had a follow-up of 27 months, with the last swab positive for meningococci 21 months after meningococcal carriage detection, showing that meningococcal carriage can persist for a long time. Previous longitudinal studies have shown varying results regarding the duration of carriage [2,29], but to our knowledge, no previous studies have followed carriers for this long. All prolonged carriers carried the same serogroup in repeated samples. Successive strains from the same carrier were closely related, with 0–3 allelic differences, indicating that prolonged carriage is probably due to persistent carriage with the same strain. No participant in the study developed invasive meningococcal disease.

The absolute and relative abundance of *N. meningitidis* was not higher in the first sample from prolonged carriers compared with samples from non-prolonged carriers, although the power of the comparison was limited since the non-prolonged carriers were only three after those lost to follow-up were excluded. However, comparing the absolute and relative abundance of meningococci in 35 baseline samples to 43 follow-up samples, both absolute and relative abundance were higher in follow-up samples, indicating increased abundance among prolonged carriers. In a recent, large study from Australia, increased carriage density in the first sample was associated with increased odds of persistent carriage [29].

Of the 14 sequenced strains, nine were of ST-213/CC-213, of which six were MenB and three were non-groupable with a B backbone. Eight of 10 strains from prolonged carriers belonged to the same CC (CC-213), as well as one strain from a non-prolonged carrier. This CC may have a better ability to persist than other CCs, or the high proportion in the prolonged carriers could represent its dominance in colonisation among adolescents and young adults in Iceland. Of note, all CCs identified in carriage strains in our study have been reported as the most common CCs in IMD [30,31].

This study has some limitations. Firstly, we did not collect social information about participants, but social factors have previously been shown to be risk factors for meningococcal colonisation among adolescents and young adults [2]. Secondly, we only included longitudinal follow-up of carriers at the first sampling time point and the follow-up ended with the first negative swab. Thus, information on acquisition or re-acquisition of meningococcal colonisation is missing. Thirdly, a considerable number of young adults were lost to follow-up. Fourthly, we did not test all culture-negative samples with qPCR. We found 1.4% of tested culture-negative samples to be qPCR-positive. If 1–2% of all culture-negative samples from the adolescents and young adults were positive, that would have resulted in an additional seven to 14 *N. meningitidis* samples, making the carriage prevalence of adolescents and young adults around 6% (5.8–6.8%) instead of 4.8%. Fifthly, the accuracy in absolute bacterial abundance cannot be guaranteed, as there might be some variation in how the samples were collected. However, trained health professionals collected all samples according to the same instructions and no samples were negative or weakly positive in 16S qPCR, indicating that there were no inadequately collected samples. Sixthly, we only sequenced the groupable strains and can therefore not assert that the same non-groupable strains are maintaining colonisation for several months. Finally, direct plating increases the chance of detecting meningococcal carriage [21], rather than using a transport medium and plating the swabs within a few hours.

The study has several strengths. The duration of follow-up was longer than in most studies, giving a more comprehensive view of the duration of meningococcal carriage. Molecular methods were used to determine meningococcal serogroups. We acquired information on the vaccination status of the participants in the study instead of using the vaccination uptake in the community as a reference, allowing us to better assess the effects of meningococcal vaccinations on the carriage on an individual level. Furthermore, we collected information on antibiotic use before the first swab and between swabs for prolonged carriers, which was helpful in distinguishing between natural clearance of carriage or clearance due to antibiotic use.

## Conclusion

Carriage of groupable meningococci is uncommon among children, adolescents and young adults in Iceland. MenB are the most commonly carried groupable meningococci, although rare. Carriage of MenC was not detected, probably as a result of MenC vaccinations. The WGS revealed that prolonged carriage is due to persistent carriage of the same strains. We conclude that additional meningococcal vaccinations are not needed in Iceland at this time, considering both the low carriage rates presented in this study and, importantly, a very low incidence of invasive meningococcal disease. However, the ever-changing and unpredictable epidemiology of *N. meningitidis* must be kept in mind, and vigilance regarding the need for changes in the immunisation schedule must be maintained.
